# Cycloid psychosis as a psychiatric expression of anti‐NMDAR encephalitis. A systematic review of case reports accomplished with the authors' cooperation

**DOI:** 10.1002/brb3.1980

**Published:** 2020-12-03

**Authors:** Eloi Giné Servén, Ester Boix Quintana, Maria Martínez Ramírez, Nicolau Guanyabens Buscà, Desiree Muriana Batiste, Mar Guasp, Cristina Torres Rivas, Eva Davi Loscos, Virginia Casado Ruiz

**Affiliations:** ^1^ Psychiatry Department, Hospital de Mataró Consorci Sanitari del Maresme Mataró Spain; ^2^ Neurology Department, Hospital de Mataró Consorci Sanitari del Maresme Mataró Spain; ^3^ Neuroimmunology Program Institut d'Investigacions Biomèdiques August Pi i Sunyer (IDIBAPS) Hospital Clínic Universitat de Barcelona Barcelona Spain; ^4^ Neurology Department Hospital Clínic Barcelona Spain; ^5^ Centro de Investigación Biomédica en Red Enfermedades Raras (CIBERER) Madrid Spain

**Keywords:** acute psychosis, anti‐NMDAR encephalitis, atypical psychosis, autoimmune, first psychotic episode

## Abstract

**Objective:**

We reviewed the psychotic symptoms of anti‐NMDA receptor encephalitis (NMDARE) to differentiate its presentation from those found in a primary psychiatric disorder. We hypothesized that the cycloid psychosis (CP) phenotype would be a frequent clinical presentation in the psychiatric phase of NMDARE.

**Method:**

A systematic literature review in PubMed of all case reports published on NMDARE was performed from database inception to March 2020. We included all cases where psychotic symptoms were reported and whose diagnoses were confirmed by the presence of anti‐NMDAR antibodies in the cerebrospinal fluid (CSF). An email including a short test (CP phenotype, Perris and Brockington's criteria) was sent to all case report authors asking them to describe the psychotic symptoms.

**Results:**

We identified 335 case reports fulfilling our criteria, and the authors of 200 replied. Our analyses were based exclusively on those answers and data extracted from the articles. Median patient age was 25 years (+‐11.4), 81% were female, and 39% had an ovarian teratoma. A complete CP phenotype was identified in 175 patients (87%). These were acute psychotic episodes with a sudden onset and a fluctuating clinical pattern mostly characterized by confusion (97%), delusions (75%), hallucinations (69%), motility disturbances (87%), and mood oscillations (80%).

**Conclusion:**

The complete CP phenotype was frequently the expression of psychotic symptoms in NMDARE. We suggest that patients with a first psychotic episode who initially exhibit the CP phenotype should undergo CSF analysis to determine whether antibodies against neuronal cell surface or synaptic receptors are present to rule out a possible diagnosis of autoimmune encephalitis.

## Graphical abstract

It is of the utmost importance to precisely describe the initial psychiatric phenotype of anti‐NMDARE to perform an early diagnosis and to ameliorate prognosis. The complete cycloid psychosis phenotype was a frequent clinical presentation of anti‐NMDA receptor encephalitis cases with psychotic symptoms. This phenotype has an acute and sudden onset and a fluctuating course and is characterized by confusion, delusions, hallucinations, mood swings, and motility disturbances.

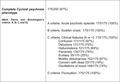

## 1 INTRODUCTION

Anti‐NMDA receptor encephalitis (NMDARE) is characterized by the presence of antibodies against the GluN1 subunit of the N‐methyl‐D‐aspartate receptor (NMDAR). This specific type of encephalitis was first identified and described in 2007 by Dalmau et al. (Dalmau et al., 2007). The diagnostic criteria (Graus et al., 2016) emphasized the need to detect specific antibodies in the cerebrospinal fluid (CSF) using specific techniques in specialized laboraotries. This ensures a correct diagnosis and reduces the number of both false positives and false negatives.

NMDARE is the second most common encephalitis (estimated incidence: 2–3 cases per million) just after acute disseminated encephalomyelitis (Granerod et al., 2010). It was found to be more common than any other individual viral etiology in young individuals (Gable et al., 2012). In a retrospective study, it represented 1% of all admissions of young adults to an intensive care unit (Pruss et al., 2010). The association between NMDARE and tumors depends on the sex and age of the individuals, with ovarian teratomas being the most frequent (Florance et al., 2009).

Approximately 70% of NMDARE patients exhibit psychiatric symptoms, mainly in the form of acute or subacute onset psychotic episodes characterized by rapid and serious evolution. Most patients do not have a previous history of psychiatric symptoms. Therefore, they are often admitted to psychiatric units with an initial diagnosis of a first psychotic episode. These episodes are usually accompanied by subtle neurological symptoms, which in most patients become more severe during the weeks that follow the initial psychiatric symptoms. These include seizures, abnormal movements, decreased levels of consciousness, or dysautonomic features. However, there is a small group of patients who only develop psychosis as a manifestation of NMDARE (Kayser et al., 2013). In general, most patients respond well to immunotherapy treatment, tumor resection (when necessary), and symptomatic monitoring, with 75%–80% achieving a significant or full recovery. Detection and early instauration of treatment during the initial psychiatric phase are critical and may improve the outcome and prognosis of these patients (Dalmau et al., 2011).

Previous studies have described the psychiatric symptoms of patients with anti‐NMDA receptor encephalitis (Al‐Diwani et al., 2019; Sarkis et al., 2019; Warren et al., 2018). For example, one systematic review article including 633 patients with NMDARE observed that 80% of them exhibited alterations in behavior, 50% experienced an alteration of language, 46% displayed symptoms of psychosis (21% delusional ideas, 31% hallucinations), 33% exhibited catatonia, and 24% experienced mood alterations (Warren et al., 2018). With respect to the evolution of the disease, a prospective study with a cohort of 571 patients diagnosed with NMDARE showed that 4% of patients exhibited isolated psychiatric symptoms (both initially and during subsequent relapses). In terms of clinical phenomenology, nearly 75% of patients exhibited symptoms of psychosis (Kayser et al., 2013).

In some of these studies, the following methodological flaws have been detected: (a) inclusion of exclusively adult patients (Al‐Diwani et al., 2019) precludes extrapolation of the results to the infanto‐juvenile population; (b) inclusion of cases where only the serum is positive (Al‐Diwani et al., 2019; Sarkis et al., 2019; Warren et al., 2018), significantly increases the number of false positive cases. Additionally, a specific clinical pattern cannot be identified. Vague descriptions of psychiatric symptomatology are made, listing the most frequent abnormal features but not providing a detailed account regarding the appearance, combination, and evolution of these symptoms. This might be because they are based on case reports written by a myriad of medical specialists (Warren et al., 2018).

In addition, with the current information available, we are not able to differentiate between psychotic symptoms that appear at the onset of NMDARE and those observed in psychosis secondary to primary psychiatric diseases.

Some studies suggest a series of warning signs that can help clinicians identify NMDARE in patients with psychotic symptoms (Herken & Prüss, 2017; Steiner et al., 2020). Many of these signs are based on the identification of neurological clinical features or abnormal test results (e.g., EEG, CSF).

To facilitate an early and accurate diagnosis of patients with isolated psychiatric symptoms, it is crucial to focus on a detailed clinical description of the psychiatric phenotype of this illness. To this end, in a previous paper, we described the psychiatric presentation of three patients with NMDARE (Giné Servén et al., 2019) and discussed the similarity of their symptomatology with that shown by patients with cycloid psychosis. This clinical entity is characterized by a sudden onset of heterogeneous symptomatology, including polymorphous psychotic symptoms, mood swings, and motility disturbance. In 1981, Perris and Brockington described the most commonly used set of criteria for cycloid psychosis (Table 1).

**TABLE 1:** Perris and Brockington's diagnostic criteria for cycloid psychosis

a) An acute psychotic episode, unrelated to substance use or to brain organicity, with an onset between 15 and 50 years of age. b) Sudden onset in a period of hours, or of a few days at most. c) To arrive at a definitive diagnosis, at least four of the following symptoms should be present: Some degree of confusion, ranging from perplexity to severe disorientation. Mood‐incongruent delusions of any kind: most often with a persecutory content. Hallucinatory experiences of any kind, often related to fear of death. An overwhelming, frightening experience of anxiety, not bound to particular situations or circumstances. Deep feelings of happiness or ecstasy, most often of a mystical nature. Akinetic or hyperkinetic motility disturbances. A particular concern with death. Background (oscillations of mood, but not pronounced enough to justify a diagnosis of an affective disorder). d) There is no fixed symptomatologic combination: on the contrary, the symptomatology may change frequently during the episode.

## 2 OBJECTIVE

We sought to characterize the psychotic symptoms that appear in anti‐NMDA receptor encephalitis through a systematic review of all published case reports. We hypothesized that the cycloid psychosis phenotype would be a frequent clinical presentation during the psychiatric phase of this autoimmune disease.

## 3 METHODS

**FIGURE 1:**
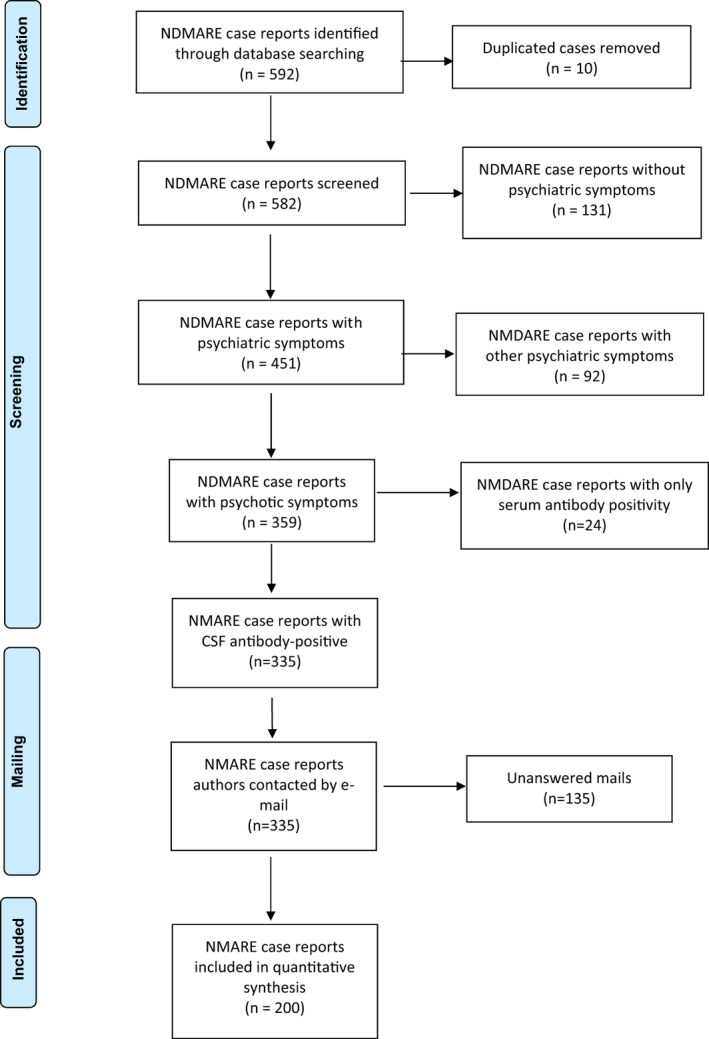
PRISMA 2009 flow diagram: published cases of anti‐NMDA receptor encephalitis. From: Moher et al. (Moher et al., 2009). For more information, visit www.prisma‐statement.org

We performed a PubMed search with the following keywords: NMDA or NMDAR or NDMARE or anti‐N‐methyl‐D‐aspartate or NMDA encephalitis or anti‐NMDAR encephalitis. Search results were limited to case reports and series written in English, Spanish, French, and German, published through March 2020 and including human subjects. Only cases with documented CSF anti‐NMDAR antibodies were included. Bibliographies of included studies were screened for additional reports. The Preferred Reporting Items for Systematic Reviews and Meta‐Analyses (PRISMA) statement recommendations were followed (Figure 1) (Moher et al., 2009). We found 592 NMDARE case reports through database search screening, and after removing duplicate publications, we selected 582 cases. From these, 451 (77.5%) included psychiatric symptomatology. We identified 335 (57.5%) case reports of CSF antibody‐positive NMDARE in which psychotic symptoms were reported. We excluded cases where only the serum was positive to avoid false positives. Afterward, an email was sent to all these case report authors with a short test (Cycloid psychosis phenotype, Perris and Brockington's criteria) to describe the psychotic symptoms. The complete cycloid psychosis phenotype was present when the patient fulfilled Perris and Brockington's criteria (A: acute psychotic episode; B: sudden onset; C: at least four of the described symptoms; D: no fixed symptomatologic combination) (Table 1).

Articles were divided equally among the four reviewers (EGS, MMR, EBQ, NGB), who used a common data extraction sheet. Each article was reviewed by one of the reviewers. The data extracted included the first author's medical specialty, demographic data, the presence of somatic or psychiatric prodromal symptoms, the identified trigger, seizures at onset or during the episode, and complementary test results (CSF routine analysis, brain MRI, EEG). Information regarding the presence or absence of anti‐NMDAR antibodies in the serum was also noted.

Descriptive statistics were employed for the initial data analysis. The prespecified subgroups (prodromal symptoms versus nonprodromal symptoms; seronegative versus seropositive patients; trigger versus nontrigger) were compared by Student's *t* test, chi‐square test, or Mann–Whitney *U* test when appropriate. Statistical significance was defined as *p < .05*. SPSS version 25 statistics software was used for the analyses.

## 4 RESULTS

From the 335 case reports selected, emails were sent to each of the authors. Two hundred of them replied, and our descriptive statistics are based exclusively on these answers and data extracted from the authors’ articles.

First, it is important to note that the authors were from many different specialties: 107 case reports were written by neurologists (53%), 40 by psychiatrists (20%), 16 by pediatricians (8%), 10 by internists (5%), 5 by gynecologists (2%), 5 by anesthesiologists (2%), 5 by emergency doctors (2%), 4 by radiologists (2%), 3 by anatomo‐pathologists (1%), 2 by ophthalmologists (1%), 2 by cardiologists (1%), and 1 by a geriatrician (0.5%).

Second, focusing specifically on the results of the answers from those 200 case reports (Table 2, Table 3), the median age of patients was 25 years (*SD* 11.4), and 163 of 200 (81%) were female. In 84 cases (42%), the trigger was identified: 78 had an ovarian teratoma (39%), 2 had a testicular seminoma (1%), and 4 had other triggers (2%). Ninety‐one cases showed somatic prodromal symptoms (45%). The most prevalent prodromal symptoms were headache (41 cases, 20%), fever (26 cases, 13%), and flu‐like syndrome (25 cases, 12%). Seizures were observed at onset or during the episode in 129 cases (64%). When going through the results of complementary studies, three major things should be pointed out: a) in CSF routine analysis, pleocytosis (>5 WBC) was described in 65% of cases (119/184); b) we found normal brain MRI in 70% of cases (131/185), but the remaining cases exhibited characteristic features (26 cases had limbic inflammatory lesions (14%), 11 cases had inflammatory/demyelinating lesions (6%) and 9 cases had nonspecific white matter lesions (4%)); and c) 82% (133/162) of EEGs were abnormal, showing diffuse slowing (37%, 60 cases), epileptic features (13%, 21 cases), extreme delta brush (9%, 15 cases), or other phenomena (23%, 37 cases).

**TABLE 2:** Summary of patients (*n* = 200)

Age (median, *SD*) [years]	25 (11.4)
Female sex (%)	163 (81%)
Somatic prodromal symptoms	91 (45%) Headache 41 (20%) Fever 26 (13%) Flu‐like syndrome 25 (12%) Gastrointestinal 15 (7%) Others 28 (14%)
Psychiatric prodromal symptoms	35 (17%)
Seizures	129 (64%)

**TABLE 3:** Complementary explorations

CSF routine analysis	Normal 65/184 (35%) Pleocytosis (>5 WBC) 119/184 (65%)
EEG	Normal 29/162 (18%) Diffuse slowing 60/162 (37%) Epileptic 21/162 (13%) Extreme delta brush 15/162 (9%) Others 37/162 (23%)
Brain MRI	Normal 131/185 (70%) Limbic inflammatory 26/185 (14%) Inflammatory/demyelinating lesions 11/185 (6%) Nonspecific white matter lesions 9/185 (4%) Other abnormalities 8/185 (4%)
NMDAR Ab LCR	200 (100%)
NMDAR Ab serum	95/111 (86%)
Seronegative patients NMDAR Ab LCR +, NMDAR Ab serum ‐	16/111 (14.4%)
Identified trigger	84 (42%) Ovarian teratoma 78 (39%) Testicular seminoma 2 (1%) Others 4 (2%)

[Correction added on January 08, 2021 after first online publication: NMDAR sèrum has been changed to NMDAR serum.]

### 4.1 Seronegative NMDARE patients

A total of 14.4% of our cases (16/111) did not exhibit serum positivity for anti‐NMDAR antibodies. These seronegative patients were less likely to have a trigger (3 [18.8%] versus 44 [46.8%]; *p* = .036). There were no other differences with respect to age, sex, presence of somatic or psychiatric prodromal symptoms, fulfillment of cycloid psychosis phenotype criteria or complementary testing (EEG, CSF analysis, or cranial MRI) between seronegative and seropositive patients.

### 4.2 Somatic prodromal phase

Patients with prodromal somatic symptoms had a higher frequency of sensory and perceptual alterations (67 [73.6%] versus 66 [60.6%]; *p* = .05) and a higher frequency of CSF pleocytosis (68 [76.5%] versus 51 [54.3%]; *p* = .003).

Stratification of patients based on a specific prodromal symptom revealed that a) patients with cephalea had a higher frequency of sensory and perceptual alterations (33 [80.5%] versus 100 [60.9%]; *p* = .033), a higher frequency of CSF pleocytosis (34 [82.9%] versus 85 [59.4%]; *p* = .006) and a higher number of cells in the CSF (73.76 versus 28.99; *p* = .000); and b) patients with fever were more concerned about death (8 [30.8%] versus 25 [14.4%]; *p* = .036) and exhibited fewer motility alterations (18 [69.2%] versus 147 [84.5%]; *p* = .056). [Correction added on January 8, 2021, after first online publication: The opening bracket have been removed.]

### 4.3 Psychosis phenotype

The complete cycloid psychosis phenotype (Perris and Brockington's criteria) was identified in 175 cases, representing 87% of total cases examined (Table 4). These were acute psychotic episodes (A criteria) with a sudden onset (B criteria) and a fluctuating clinical (D criteria) pattern mostly characterized by (C criteria) confusion (171 cases, 97%), delusions (131 cases, 75%), hallucinations (121 cases, 69%), motility disturbances (152 cases, 87%), pananxiety (130 cases, 74%), and mood oscillations (140 cases, 80%). Other symptoms were less frequent and included ecstasy (35 cases, 20%) or concern about death (33 cases, 19%).

**TABLE 4:** Anti‐NMDA receptor encephalitis patients with complete cycloid psychosis phenotype

Complete cycloid psychosis phenotype (Meet Perris and Brockington's criteria: A, B, C, and D)	175/200 (87%) A criteria: Acute psychotic episode: 175/175 (100%) B criteria: Sudden onset: 175/175 (100%) C criteria: Clinical features [4 or >]: 175/175 (100%) Confusion 171/175 (97%) Delusions 131/175 (75%) Hallucinations 121/175 (69%) pananxiety 130/175 (74%) Ecstasy 35/175 (20%) Motility disturbances 152/175 (87%) Concern with dead 33/175 (19%) mood oscillations 140/175 (80%) D criteria: Fluctuation: 175/175 (100%)

[Correction added on January 8, 2021 after first online publication: Value of mood oscillation has been changed.]

In the group of NMDARE patients who did not fulfill all Cycloid Psychosis criteria (*n* = 25), 13 cases (52%) exhibited an acute presentation (A criteria), 10 cases (40%) experienced a sudden onset (B criteria), 10 cases (40%) had fluctuations (D criteria), and some fulfilled only the C criteria, including 22 cases (88%) with confusion, 12 cases (48%) with hallucinations, 7 cases (28%) with delusions, 13 cases (52%) with motility disturbances, and 9 cases (36%) with mood oscillations.

Only 35 patients (17%) had psychiatric prodromal symptoms, such as personality changes or negative symptoms, usually lasting a few weeks. This subgroup of patients seems to have a different clinical pattern characterized by reduced confusion (30 [85.7%] versus 163 [98%]; *p* = .000), increased delusional ideas (29 [82.9%] versus 109 [66.1%]; *p* = .051), and fewer fluctuations in their symptomatology (29 [82.9%] versus 156 [94.5%]; *p* = .017).

## 5 CONCLUSIONS

It is very important to precisely describe the psychiatric symptomatology that appears during the initial phase of NMDARE to perform precocious detection and to initiate treatment as soon as possible to ameliorate the prognosis. Until now, previous systematic revisions versing the psychiatric symptomatology of NMDARE have been unable to establish a characteristic pattern to help clinicians identify the condition early in the disease course or to differentiate the psychiatric presentation of NMDARE from a primary psychiatric illness. This difficulty is caused by the lack of good descriptions of psychiatric symptomatology in the vast majority of case reports that are used to performing systematic revisions. They miss information about the appearance, combination, and evolution of these symptoms.

To identify a pattern of psychotic symptoms that are usually observed in NMDARE, we performed a systematic revision including patients from all ages and excluding those with positivity only in serum samples to avoid false positives. We contacted each of the authors of the selected articles to evaluate our hypothesis based on detailed clinical observations of our patients (Giné Servén et al., 2019): The cycloid psychosis phenotype might be a clinical expression frequently found in NMDARE patients. From those initial 335 cases, 200 authors replied, and we based our analyses exclusively on their answers and publications.

The data from our selected sample were similar to those reported in a previous reliable study (Guasp et al., 2020; Kayser et al., 2013), indicating that our sample is representative of the disease. This is observed in categories such as demographic data (median age = 25, 81% female), association with tumors (39% ovarian teratoma), presence of seizures (60%), and complementary testing (LCR pleocytosis 65%, normal brain MRI 70%, abnormal EEG 82%). Seronegative patients (16/111; 14.4%) exhibited lower tumoral frequency (3 [18.8%] versus 44 [46.3%]; *p* = .039), in accordance with previous studies (Guasp et al., 2020).

The presence and type of somatic prodromes were related to the expression of specific psychiatric symptoms. Cephalea is related to a higher presence of sensory and perceptual alterations, as well as fever with a lower frequency of motility changes and a greater concern with death. To our knowledge, these findings have not been previously described.

However, the most relevant result of our review, which is in concordance with our initial hypothesis, is that of a total of 200 answers, 87% of patients fulfilled the complete cycloid psychosis phenotype in the psychiatric phase of NMDARE, according to the criteria of Perris and Brockington.

The concept of cycloid psychosis dates back to the work of Bénédict Morel in 1857 (Morel, 1857) and was further developed by Valentin Magnan in 1895 (Magnan, 1895). This latter author was the first to publish a scientific paper identifying a clinical entity characterized by sudden onset of polymorphous psychotic symptomatology. The existence of this clinical entity gained widespread acceptance in Germany, where the term cycloid psychosis was coined. Nevertheless, these clinical characteristics (or very similar ones) have been described by other authors and/or schools and have been given a number of names, including acute schizoaffective psychosis (The acute schizoaffective psychoses, 1994), atypical psychosis (Mitsuda, 1965), postpartum psychosis (McNeil, 1986), and Bouffée délirante (Pichot, 1982). Ultimately, the term cycloid psychosis became predominant because it was developed within the framework of a coherent and well‐integrated classification system (Cuesta & Peralta, 2007). In 1981, Perris and Brockington drafted the first operational set of diagnostic criteria to define this clinical entity, and these remain the most commonly used criteria today (Brockington et al., 1982; Perris, 1974). This clinical entity is more common in young women and has been frequently related to seizures. In a study of 92 cases of cycloid psychosis written in 1982 (Barcia, 1982), 15 patients had a history of seizures prior to the appearance of psychotic symptoms, 19 patients were suffering seizures when the psychotic symptoms began, and 7 other patients with no seizures exhibited hypersynchrony in their EEG with temporal focus, suggesting comiacility. A total of 51.4% of all EEGs in this study were irregular with “temporolimbic paroxistic dysrhythmias”.

The link we established between this classic entity and NMDARE may generate hypotheses in two different directions:

On the one hand, it could be that a subgroup of patients who in the past were diagnosed with cycloid psychosis, especially the most severe cases that required ECT or had seizures or abnormal EEG, were in fact NMDAREs that went undiagnosed.

On the other hand, the cycloid psychosis phenotype in a first psychotic episode raises suspicion of a possible NMDARE. Therefore, we suggest that these patients undergo a lumbar puncture to determine the presence or absence of antibodies in the CSF to rule out the possibility of NMDARE. To investigate whether this clinical marker might be useful for detecting NMDARE during the initial phase, it would be necessary to perform prospective comparative studies in which patients presenting a first episode of psychosis are duly evaluated (e.g., analysis of determination of anti‐NMDAR antibodies in the CSF).

There are a number of limitations inherent in the methodology of this study that should be considered. Case reports and case series are subject to reporting and publication bias. These results are not valid in children under the age of fifteen due to the age span of patients from the case reports we included in the study. In addition, response bias should also be noted: it could be possible that only authors who identified this clinical pattern in their patients answered our message.

## CONFLICT OF INTEREST

None declared.

## AUTHOR CONTRIBUTION

EGS designed, hypothesized, drafted, and revised the manuscript. EBQ designed and drafted the manuscript. MMR drafted and revised the manuscript. NGB revised the manuscript. DMB revised the manuscript. MG: revised the manuscript. CTR revised the manuscript. EDL revised the manuscript. VCR drafted and revised the manuscript.

### Peer Review

The peer review history for this article is available at https://publons.com/publon/10.1002/brb3.1980.
